# Atomic level three-dimensional structure of individual particles with XFELs

**DOI:** 10.1107/S2052252518011909

**Published:** 2018-09-01

**Authors:** Changyong Song

**Affiliations:** a Department of Physics, Pohang University of Science and Technology, Pohang 37673, Republic of Korea

**Keywords:** single-particle three-dimensional imaging, X-ray free electron lasers, imaging virus particles, image analysis, three-dimensional reconstruction, atomic scale structure

## Abstract

Progress in single-particle three-dimensional imaging is discussed, with advances in both data-collection and data-handling techniques described.

Interest in acquiring three-dimensional atomic structures using single specimens has been extremely keen. The possibility of bypassing the painstaking effort to make crystals is a huge attraction and has invigorated single-particle three-dimensional imaging research, with remarkable accomplishments in unveiling the structures of many challenging proteins and macromolecular complexes using cryo-electron microscopy (cryoEM) (Renaud *et al.*, 2018 ▸). The cryoEM technique has been an unrivalled method of choice for atomic level single-particle three-dimensional imaging.

The X-ray free-electron laser (XFEL) is changing this landscape, as XFEL single-pulse imaging joins the race (Ekeberg *et al.*, 2015 ▸; Martin & Loh, 2013 ▸). Applications of femtosecond X-ray laser pulses from XFELs for single-particle imaging introduce a complementary capability to image specimens under ambient conditions, preserving native sample conditions and with a natural extension to follow dynamics (Spence, 2017 ▸). XFEL single-particle imaging is realized by collecting a large number of coherent diffraction patterns from single specimens of high homogeneity using single XFEL pulses. As each diffraction pattern represents the projected electron density of the specimen at a random orientation, the three-dimensional orientation among the measured diffraction patterns needs to be figured out, which is usually taken care of by specially devised numerical algorithms (Gaffney & Chapman, 2007 ▸).

In this issue of **IUCrJ**, Filipe Maia and colleagues (Lundholm *et al.*, 2018 ▸) report the three-dimensional reconstruction of Melbourne virus (MelV) *via* XFEL single-particle diffraction imaging, while systematically investigating the data-handling process and its propagation to a reconstructed three-dimensional image. Coherent diffraction patterns were collected at the Linac Coherent Light Source (LCLS, Stanford, California, USA) from single MelV particles with the nominal size of 230 nm in diameter using 0.1 GW X-ray pulses on the samples, delivering ∼10^11^ photons of 1.2 keV photon energy. Virus particles were delivered to the X-ray interaction spot through an aerosol injector. The chance of hitting a single virus particle with a pulsed X-ray beam is stochastic, governed by Poisson statistics (Park *et al.*, 2013 ▸). Out of 965 739 patterns containing scattered X-ray photons, only 260 patterns were selected as displaying valid diffraction signals of single virus particles with good sample homogeneity.

These 260 two-dimensional patterns at random orientations were assembled into a three-dimensional diffraction pattern using the EMC algorithm. This algorithm performs the three-dimensional assembly by maximizing the likelihood between conjectured and measured diffraction patterns while searching over an expanded parameter space (Loh & Elser, 2009 ▸; Fung *et al.*, 2008 ▸).

Finally, a three-dimensional image of the virus particle was obtained at 28 nm resolution by retrieving the phase of the oversampled three-dimensional coherent diffraction patterns through iterative operations of discrete Fourier transformation between real and diffraction space (Miao *et al.*, 1999 ▸).

The process appears straightforward, but the caveat is in accepting all the details of the obtained three-dimensional image as real features, even though they can result from experimental noise. The authors have demonstrated the crucial importance of eliminating background noise, which produces an artificial high-density structure at the image center. Further, the influence of sample inhomogeneity, intensity fluctuation, algorithm-specific parameters *etc.* on the three-dimensional image were studied to provide practical guidelines for three-dimensional single-particle imaging *via* XFELs.

The research field of XFEL single-particle three-dimensional imaging is immature but expanding fast. Available data are yet scant and more experimental data need to be collected, which will help to refine experimental techniques and data-handling protocols. Proof-of-principle results showing three-dimensional images have been reported (Ekeberg *et al.*, 2015 ▸; Kurta *et al.*, 2017 ▸). However, experimental data from controlled systems are still needed to evaluate the fidelity of the reconstructed images under given experimental conditions.

The interest in XFEL dynamic single-particle imaging is significant, stimulating research to introduce advanced schemes for sample delivery and data processing. Given the increasing availability of XFEL sources (Kang *et al.*, 2017 ▸) and higher repetition rate operations on the megahertz scale, we expect to observe many new research activities in single-particle three-dimensional imaging with increased image resolution to address challenging scientific issues of interest.

## Figures and Tables

**Figure 1:**
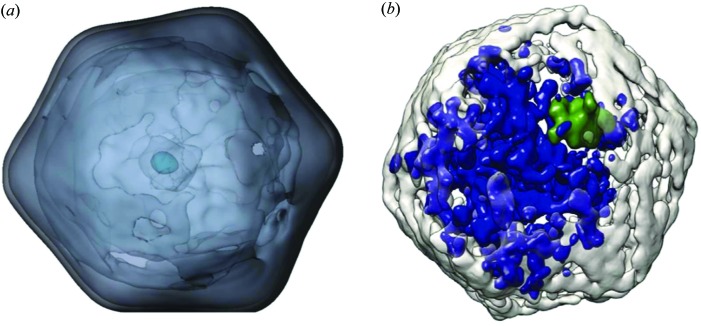
Single-particle three-dimensional imaging of Melbourne virus (MelV). (*a*) A reconstructed image from XFEL single-particle three-dimensional imaging. (*b*) An MelV image obtained from cryoEM tomographic reconstruction for comparison with panel (*a*). Reproduced with permission from Okamoto *et al.* (2018 ▸) under a Creative Commons Attribution 4.0 International License, https://creativecommons.org/licenses/by/4.0/.
